# P02 Assessment of tedizolid susceptibility against a collection of MRSA and VRE in comparison with linezolid

**DOI:** 10.1093/jacamr/dlag102.008

**Published:** 2026-06-26

**Authors:** Bharat Chandra Das, Md Nizam Ahmed, Madhavi Kirti, Aarsi, Parul Singh, Purva Mathur

**Affiliations:** Microbiology Unit, Jai Prakash Narayan Apex Trauma Centre, AIIMS, New Delhi; Microbiology Unit, Jai Prakash Narayan Apex Trauma Centre, AIIMS, New Delhi; Microbiology Unit, Jai Prakash Narayan Apex Trauma Centre, AIIMS, New Delhi; Microbiology Unit, Jai Prakash Narayan Apex Trauma Centre, AIIMS, New Delhi; Microbiology Unit, Jai Prakash Narayan Apex Trauma Centre, AIIMS, New Delhi; Microbiology Unit, Jai Prakash Narayan Apex Trauma Centre, AIIMS, New Delhi

## Abstract

**Background:**

MDR Gram-positive bacterial infections remain a major therapeutic challenge in high-acuity trauma settings. MRSA and VRE are frequent causes of skin and soft tissue infections, nosocomial infections and bacteraemia. The emergence of linezolid resistance has further restricted available options.

**Objective:**

To evaluate the susceptibility of tedizolid, a newer oxazolidinone, against VRE and MRSA isolates. Data from India regarding its efficacy is lacking.

**Material and methods:**

A prospective surveillance study was conducted on 103 non-duplicate MRSA and VRE isolates, obtained between 2023 and 2025 from a level-1 trauma centre in India. The collection included 53 MRSA and 50 VRE isolates. Susceptibility testing for VRE and MRSA were performed using gradient diffusion Etest strips. MRSA isolates were also tested by using Kirby-Bauer disc-diffusion method. The breakpoints are based on recent CLSI guidelines. Linezolid (30μg disc) and tedizolid (2 μg disc) were used. For quality control (QC) *Staphylococcus aureus* (ATCC 25923) was used and *Enterococcus faecalis* (ATCC 29212) was used as a supplemental strain. By using densitometer,.5 McFarland standard density of test organisms was made. Lawn culture was done according to Kirby-Bauer disc diffusion method. The tedizolid Etest strip was kept in the middle of the lawn culture and gently pressed. Categorical agreement between linezolid and tedizolid results was analysed using Cohen’s Kappa and sensitivity, specificity, and positive predictive value (PPV), negative predictive value (NPV) were calculated.

**Results:**

Drug susceptibility tests revealed that 7 linezolid-resistant VRE isolates had low (≤.5 mg/L) tedizolid MIC. Two tedizolid resistant VRE strain showed susceptibility against linezolid. Tedizolid demonstrated almost perfect agreement with linezolid among VRE isolates (Cohen’s κ=0.852), with 95% sensitivity, 100% specificity, 100% PPV, and 77.8% NPV. Among 53 MRSA isolates 3 isolates were linezolid resistant which were also resistant against tedizolid. By E strip method, there was 100% concordance between linezolid and tedizolid susceptibility of MRSA isolates. Two out of 50 linezolid susceptible MRSA isolates were resistant against tedizolid by disc diffusion method. Cohen’s kappa value showed substantial agreement of the disc diffusion method for tedizolid and linezolid (kappa=0.731) with sensitivity of 96%, specificity and PPV of 100, NPV of 60%.

**Conclusions:**

Tedizolid exhibited potent in vitro activity against MRSA and VRE isolates, collected from a high-burden trauma centre. Importantly, it retained activity against the majority of linezolid-resistant VRE isolates, supporting its role as a therapeutic option where linezolid resistance is encountered. In view of low NPV of detecting susceptibility of MRSA against tedizolid by disc diffusion method is a concern. Perfect agreement between e strip method can be used in difficult scenarios. To our knowledge, this is the first study to report tedizolid susceptibility in VRE and MRSA from India, addressing a critical gap in regional antimicrobial resistance surveillance.

